# Structure-Forming Corals and Sponges and Their Use as Fish Habitat in Bering Sea Submarine Canyons

**DOI:** 10.1371/journal.pone.0033885

**Published:** 2012-03-21

**Authors:** Robert J. Miller, John Hocevar, Robert P. Stone, Dmitry V. Fedorov

**Affiliations:** 1 Marine Science Institute, University of California Santa Barbara, Santa Barbara, California, United States of America; 2 Greenpeace USA, Washington D.C., United States of America; 3 Auke Bay Laboratories, Alaska Fisheries Science Center, National Marine Fisheries Service, Juneau, Alaska, United States of America; 4 Center for Bio-Image Informatics, Vision Research Lab, Electrical and Computer Engineering, University of California Santa Barbara, Santa Barbara, California, United States of America; Heriot-Watt University, United Kingdom

## Abstract

Continental margins are dynamic, heterogeneous settings that can include canyons, seamounts, and banks. Two of the largest canyons in the world, Zhemchug and Pribilof, cut into the edge of the continental shelf in the southeastern Bering Sea. Here currents and upwelling interact to produce a highly productive area, termed the Green Belt, that supports an abundance of fishes and squids as well as birds and marine mammals. We show that in some areas the floor of these canyons harbors high densities of gorgonian and pennatulacean corals and sponges, likely due to enhanced surface productivity, benthic currents and seafloor topography. Rockfishes, including the commercially important Pacific ocean perch, *Sebastes alutus*, were associated with corals and sponges as well as with isolated boulders. Sculpins, poachers and pleuronectid flounders were also associated with corals in Pribilof Canyon, where corals were most abundant. Fishes likely use corals and sponges as sources of vertical relief, which may harbor prey as well as provide shelter from predators. Boulders may be equivalent habitat in this regard, but are sparse in the canyons, strongly suggesting that biogenic structure is important fish habitat. Evidence of disturbance to the benthos from fishing activities was observed in these remote canyons. Bottom trawling and other benthic fishing gear has been shown to damage corals and sponges that may be very slow to recover from such disturbance. Regulation of these destructive practices is key to conservation of benthic habitats in these canyons and the ecosystem services they provide.

## Introduction

Autogenic ecosystem engineers, such as trees or corals, modify habitats through their physical presence [1]. Ecosystem engineers can provide living space [2], [3], alter and ameliorate physical conditions [4], [5] and affect biological interactions [6], potentially enhancing diversity and changing patterns of species composition and dominance on local or landscape scales [5]. In the oceans, hermatypic corals are archetypical examples of autogenic engineers, forming massive reefs in shallow tropical waters that support >30% of described marine species [7].

In deeper water, the role of corals and sponges as autogenic engineers is not well understood; nevertheless, many fishes and macroinvertebrates inhabit deep-water coral [8], [9], [10], [11] and sponge [12], [13] habitats. Unlike shallow-water systems, where the processes connecting habitat with populations and communities of dependant organisms are relatively well understood, the role of habitat structure, including that of autogenic ecosystem engineers, in deep-water communities is still unclear due to lack of small-scale observational and experimental studies [14]. Biogenic structure can affect populations of associated animals through habitat selection at settlement, differential survival, and post-settlement migration [15], [16], [17]. The result of these processes is elevated abundances of benefited organisms associated with the habitat feature in question. This conceptual framework is formalized in frequency-dependent habitat selection models [18]; e.g. the ideal free distribution model predicts positive correlation between abundance and habitat fitness value [19], [20]. Comparison of species distribution patterns with respect to potential functionally equivalent habitats, e.g. corals versus rock outcrops, can provide a better indication of the importance of autogenic engineers to associated species compared with simple observations of high densities around corals [20]. Comparative approaches, no matter how well designed, will not shed much light on mechanisms of habitat effects on associated organisms; nevertheless such approaches are crucial for informing managers and designing future experimental work.

Slow growing fragile corals are highly vulnerable to damage by physical contact with fishing gear [11], [21], [22], [23] leading the North Pacific Fishery Management Council (NPFMC) to identify gorgonian corals as essential fish habitat of particular concern [24]. Two general concepts motivating conservation of deep-water corals have been put forth: (1) corals are used as habitat by, and presumably provide some important benefit to economically important fishes and crustaceans [11], [25], [26] and (2) corals have intrinsic value, are slow growing and extremely sensitive to disturbance, and consequently have questionable potential for recovery [20]. Nevertheless, information is lacking on distribution of corals and their use as fish habitat off Alaska (but see [11], [27]), and the NPFMC has identified research on this topic as a critical need for fisheries management [28]. Here we evaluate density of structure-forming corals and sponges in Zhemchug and Pribilof Canyons, Bering Sea. We also measured densities of eight common taxa of demersal fishes, and evaluated their use of corals, sponges, and boulders as habitat in the canyons. We document and describe fishing damage to benthic habitats observed during our surveys.

## Methods

### Study sites

Zhemchug and Pribilof Canyons are enormous submarine canyons cutting into the continental slope under the northeastern boundary of the Aleutian Basin, southeastern Bering Sea. Volumes of Zhemchug and Pribilof canyons are 8500 and 1300 km^3^, respectively, and Zhemchug is likely the world's largest submarine canyon (for comparison, Monterey Canyon off California, often described as large, has a volume of only 450 km^3^) [29]. These canyons were probably excavated in the Pleistocene by mass wasting, slumping and creeping of sediment that accumulated at the heads of the Yukon and Kuskokwim rivers during periods of glacially lowered sea level [29], [30], [31]. Pribilof Canyon is ∼150 km long and ∼1500 m deep where it cuts the shelf, descending to the continental rise at ∼3000 m depth. Zhemchug Canyon is ∼160 km long, up to 2600 m deep, and intersects the continental Rise at 3400 m depth. The slopes of both canyons are largely composed of clay and marine-derived sediments [29], [32]. Detailed geological descriptions of the canyons may be found in Scholl et al. [29]. Currents in the canyons are generally moderate, ∼2–18 cm s^−1^, and follow canyon topography [33]; however, these were measured at 50 m depth, and near bottom currents may be considerably stronger at times [34].

### Video transects from submersible dives

**Figure 1:**
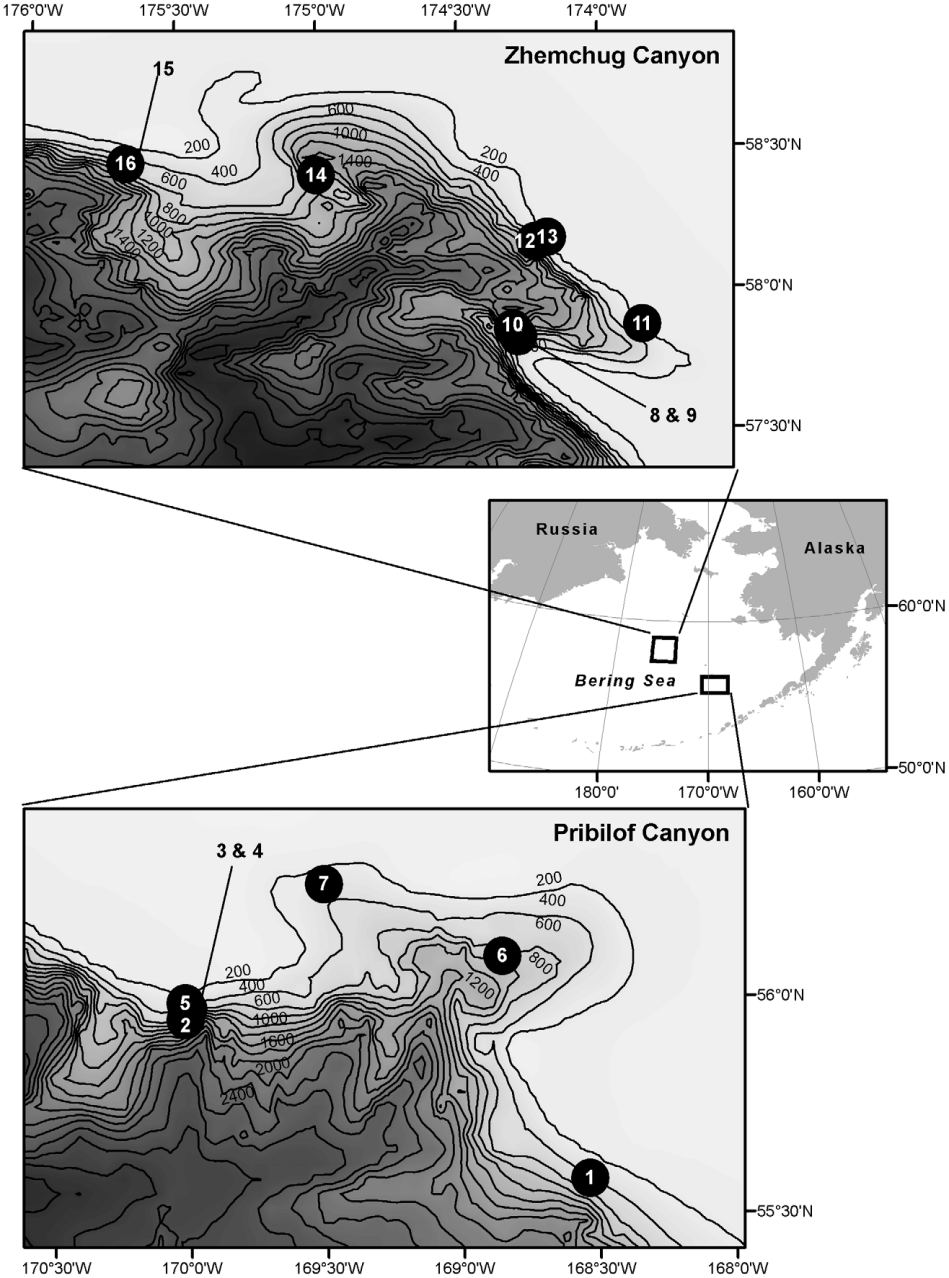
Map of study area showing locations of canyons and submersible transects.

Video transects were conducted in Zhemchug and Pribilof Canyons, Bering Sea (see map Figure 1), in Jul-Aug 2007 using Deep-Worker submersibles at depths of 168–533 m (n = 7 transects for Pribilof, n = 9 for Zhemchug). Deep-Workers are small, single-person piloted submersibles with maximum operating depth of 600 m. Transects were located to cover the geographical extent of the canyons and were located approximately equidistantly apart. Pilots flew upslope on a constant heading, with video camera (Sony HDR FX1) on the widest lens setting (58° horizontal and 32° vertical angle of view) and positioned at 30° downward from horizontal. Paired lasers 10 cm apart were projected onto the seafloor approximately in the center of the image and were used as a scale reference. Time and depth were recorded and cross-referenced to the video frames. Additional dives were done with the Deep-Worker submersibles specifically to collect specimens, and a remotely operated vehicle (ROV) was deployed to explore deeper habitats. Video footage from neither of these sources was used to quantify organism densities for this manuscript. Evidence of fishing impacts was recorded on these additional dives.

### Data analysis

Non-overlapping frames were extracted from each video transect at a constant frequency of 1 frame per 30 s using open source utility software (Bio-Image Converter [35],). An image-processing algorithm developed in Matlab, using adaptive thresholding, connected component analysis, and the CIELAB color space was used to locate the laser dots. Each frame was then manually annotated using open-source software (Scientist's Digital Notebook [36],). To ensure the quality of each frame, we checked overall scene quality, presence of overlap with adjacent images, and accuracy of automated laser dot detection. Final annotations comprise locations of laser dots and all objects of interest in the frame, and are stored in a hierarchical XML format within the Bisque system [37]. Each object was described by a location centroid and a type, which was a taxon or other identifier. We annotated a total of 54 object types, including taxa and bottom features, using an annotation template for Digital Notebook (see Text S1, Figure S1). These count data were compiled into comma-separated values files for analysis.

The area of each frame was estimated using a geometrical approach. Physical pixel resolution was computed based on the laser dots, and the area was projected from the image plane to the seabed, assuming that the seabed underneath the camera was planar [38]. Corals, sponges, and fishes were enumerated in each frame. Corals and sponges larger than ∼5 cm height with their base in the frame were counted. Dominant (>50% cover) substrate type was scored in each frame following a generalized version of the Wentworth scale [39] with fine sediment categories grouped as soft sediment, and pebble categories grouped as pebbles. Obvious fishing damage, which comprised trawl scars and/or broken corals and sponges, were scored present/absent in each frame following methodology used previously in the region [11].

Image and meta data collected and annotated for this study are publicly available on the Bisque database (http://bisque.ece.ucsb.edu/client_service/view?resource=http://bisque.ece.ucsb.edu/data_service/dataset/1580770) maintained by the Center for Bio-Image Informatics at UC Santa Barbara for researchers, educators and students and providing advanced data query, visualization and summarization tools. All developed software is publicly available as Matlab and Python scripts distributed on the Center for Bio-Image Informatics web site (http://www.bioimage.ucsb.edu/).

Coral taxa were classified into two groups for data analysis: (1) gorgonians (Subclass Octocorallia, Order Gorgonacea), and (2) sea whips and sea pens (Subclass Octocorallia, Order Pennatulacea). True soft corals (Subclass Octocorallia, Order Alcyonacea) and stoloniferen corals (Subclass Octocorallia, Order Stolonifera) also occurred on some transects but were rare and not included in analyses. Sponge taxa were classified as hexactinellid sponges (Class Hexactinellida) or calcareous sponges (Class Calcarea) and demosponges (Class Demospongiae) combined into “other sponges.”

Associations of fishes with structure-forming biota (corals and sponges) and non-biogenic structure (boulders) were evaluated using logistic regression [Generalized linear model (GLM), binomial distribution with logit link] on presence/absence frame-specific data. The response variable (fish presence) was coded 0–1 (presence-absence) as were the covariates corals and sponges, to avoid giving too much weight to frames containing numerous fishes and corals. The null hypothesis for these analyses was of the form: fish sp. A was no more likely to occur in frames containing coral than in frames not containing coral. The generalized linear model was fit to the data by Firth-adjusted maximum likelihood estimation of the parameter vector. We used Firth-penalized maximum likelihood adjustment to minimize any possible effect of collinearity and small sample sizes on the results; non-Firth-adjusted values were very similar, suggesting that these issues were not a problem in the data. Model parameters were estimated numerically through an iterative fitting process. Statistical analyses were performed in JMP (SAS Institute, version 8.0.1).

## Results

**Figure 2:**
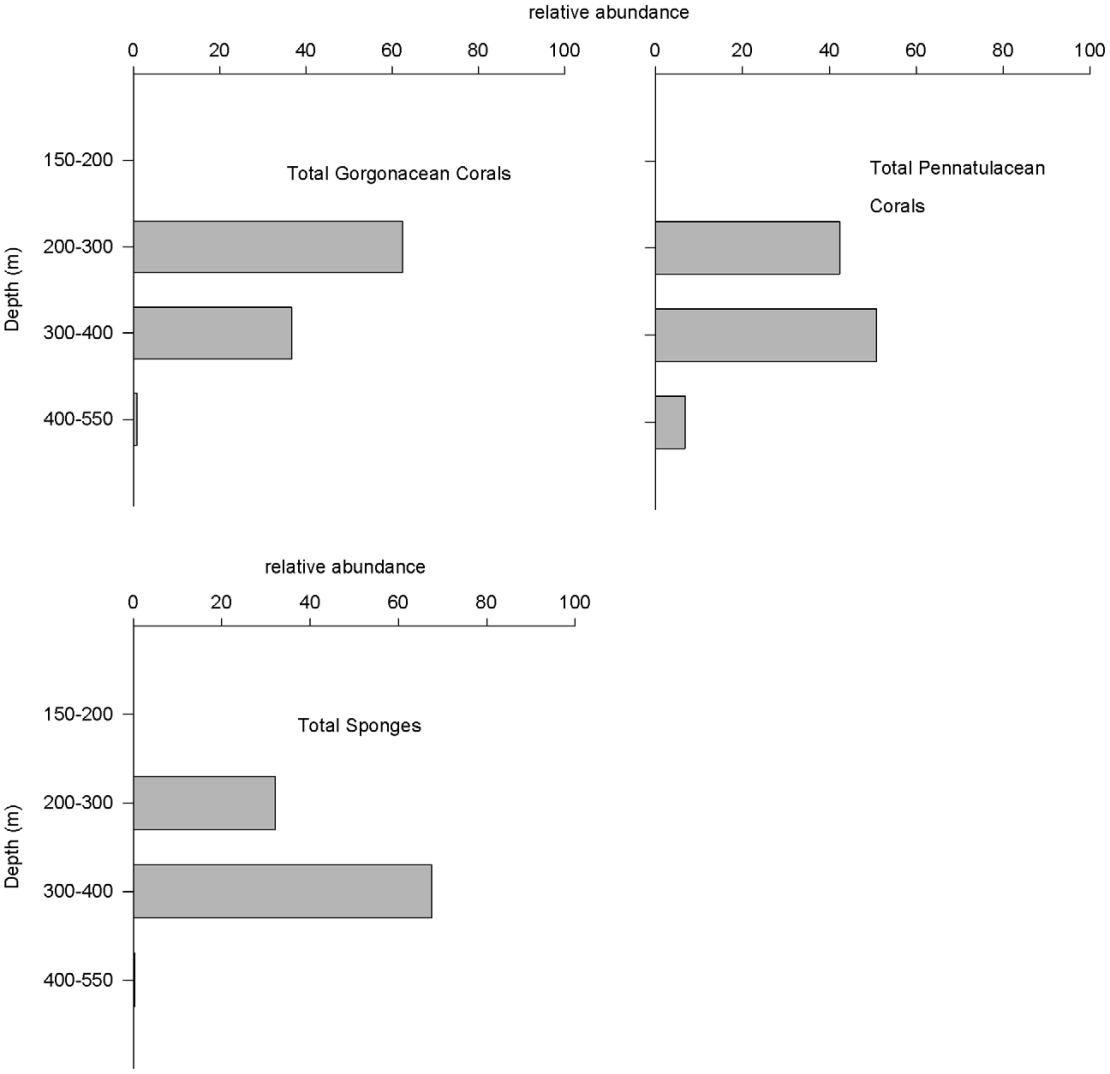
Relative abundance by depth of corals (total gorgonians n = 1301, total pennatulaceans n = 552) and total sponges (n = 1039) in Pribilof and Zhemchug Canyons, Bering Sea. Sample sizes (# images analyzed) for each depth strata were: 150–200: n = 77, 200–300: n = 483, 300–400: n = 396, 400–550: n = 1872.

A total of 2,753 frames from 16 dives were analyzed, representing a total of 23.3 hours (884GB) of high definition video. The total area sampled was ∼4202 m^2^ (1528 m^2^ in Pribilof, 2674 m^2^ in Zhemchug). Area of frames averaged 1.5 m^2^ (^±^0.02 S.E.). Transects in both canyons were dominated by soft sediment substrate (mean 65.6% cover ^±^15 S.E. for Pribilof Canyon, 85.4%^±^9 for Zhemchug Canyon), with lesser coverage of pebbles (32.3% cover ^±^15 S.E. for Pribilof Canyon, 4.1%^±^4 for Zhemchug Canyon), and cobble/boulder field (6.3% cover ^±^3 S.E. for Pribilof Canyon, 11.4%^±^7.5 for Zhemchug Canyon). Depth of transects ranged from 168 to 533 m; average depth of Pribilof transects was 306 m (^±^2 S.E., range 168–417 m), while Zhemchug transects were deeper on average, 455 m (^±^1 S.E., range 351–533 m); this difference was significant (t = 4.9, df = 14, P<0.001). Gorgonacean corals (*Plumarella superba*, *P*. *echinata*, and *Swiftia pacifica*) were associated with pebble and cobble bottoms and were ∼6× more abundant on the Pribilof Canyon transects (Table 1, transect means 0–2.41 gorgonians m^−2^, compared to 0–0.96 gorgonians m^−2^ in Zhemchug Canyon transects). Sponges were also associated with hard substrate and were ∼20× more abundant in Pribilof Canyon compared with Zhemchug (Table 1). Hexactinellid sponges were more abundant than other sponge taxa at both canyons. Pennatulacean corals (*Halipteris willemoesi* and *Protoptilum* sp.), however, were associated with fine sediment, and were still ∼5× more abundant on Pribilof transects (Table 1). Overall, corals were ∼5× more abundant on Pribilof Canyon transects. Gorgonian corals and sponges were most abundant between 200–300 m depth, whereas pennatulacean corals were most abundant between 200–400 m depth (Figure 2).

**Table 1:** Mean density estimates (numbers m^−2^^±^ S.E.) and depth range of common corals and sponges in Pribilof and Zhemchug Canyons, Bering Sea.

Taxon	Pribilof Canyon	Zhemchug Canyon	Depth range (m)
Gorgonacea			
*Plumarella* spp.	0.72 (^±^0.4)	0	237–356
*Swiftia pacifica*	0	0.08 (^±^0.1)	351–530
*Keratoisis* sp.	0.01 (^±^0.01)	0.05 (^±^0.1)	466–533
Total gorgonians	0.73 (^±^0.4)	0.13 (^±^0.1)	237–533
Pennatulacea			
*Protoptilum* sp.	0.17 (^±^0.1)	0.04 (^±^0.02)	185–529
*Halipteris willemoisi*	0.07 (^±^0.1)	0.001 (^±^0.001)	254–488
Total pennatulaceans	0.24 (^±^0.2)	0.05 (^±^0.02)	185–529
Total corals	0.97 (^±^0.4)	0.18 (^±^0.1)	
Porifera			
Hexactinellidae	0.40 (^±^0.3)	0.02 (^±^0.01)	241–466
Other sponges	0.24 (^±^0.2)	0.001 (^±^0.002)	201–306
Total sponges	0.41 (^±^0.4)	0.02 (^±^0.01)	201–466

Means are based on estimated transect densities, n = 7 transects for Pribilof, n = 9 for Zhemchug.

Commercially important Pacific ocean perch, *Sebastes alutus*, were more abundant by two orders of magnitude on Pribilof Canyon transects compared to Zhemchug Canyon (Table 2), and total rockfish abundance was likewise much higher in Pribilof Canyon (Table 2). Rajids (skates), cottids (sculpins), and zoarcids (eelpouts) were also more abundant on Pribilof Canyon transects (Table 2). Abundance of agonids (poachers), and the giant grenadier, *Albatrossia pectoralis*, was higher in Zhemchug Canyon (Table 2). Densities of pleuronectid flounders were similar in transects at both canyons. Less common fishes counted included *Gadus macrocephalus* (Pacific cod), *Anoplopoma fimbria* (sablefish), *Zaprora silenus* (prowfish), and liparids (snailfishes).

**Table 2:** Mean density estimates (numbers m^−2^^±^ S.E.) of common fishes in Pribilof and Zhemchug Canyons, Bering Sea.

Taxon	Pribilof Canyon	Zhemchug Canyon
Scorpaenidae		
*Sebastes alutus*	0.11 (^±^0.03)	0.002 (^±^0.001)
*Sebastes* spp.	0	0.02 (^±^0.02)
*Sebastolobus alascanus*	0.07 (^±^0.1)	0.02 (^±^0.01)
Total rockfish	0.18 (^±^0.1)	0.04 (^±^0.02)
Agonidae (poachers)	0.01 (^±^0.003)	0.05 (^±^0.02)
Cottidae (sculpins)	0.01 (^±^0.004)	0.002 (^±^0.001)
Macrouridae (grenadiers)		
*Albatrossia pectoralis*	0	0.004 (^±^0.002)
Pleuronectidae (right-eyed flounders)	0.02 (^±^0.01)	0.02 (^±^0.004)
Rajidae (skates)	0.01 (^±^0.004)	0.004 (^±^0.001)
Zoarcidae (eelpout)	0.01 (^±^0.01)	0.002 (^±^0.001)

*Sebastes* spp. includes *S. borealis*, the shortraker rockfish, and *S. aleutianus*, the rougheye rockfish.

Means are based on estimated transect densities, n = 7 transects for Pribilof Canyon, n = 9 for Zhemchug Canyon.

**Figure 3:**
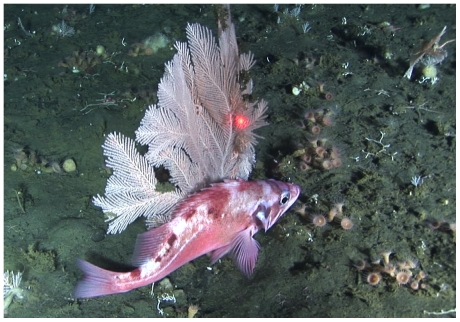
Pacific ocean perch (*Sebastes alutus*) with the gorgonian coral *Plumarella* sp. at a depth of 230 m in Pribilof Canyon, Bering Sea.

Because the depth of transects was significantly different between canyons, we analyzed the associations of fishes with structure separately for each canyon. In both canyons, rockfish (combined) were significantly more likely to be encountered near boulders and gorgonian corals (Table 3). In Zhemchug Canyon, rockfish were also more likely to be encountered near pennatulacean corals. Pacific ocean perch were significantly associated with boulders, sponges, and gorgonian corals in Pribilof Canyon, where this species was most abundant (Figure 3). In Zhemchug canyon, Pacific ocean perch were significantly associated with pennatulacean corals, but not with boulders and gorgonians, which were less abundant there (Table 3). *Sebastes* spp. (S. *borealis*, the shortraker rockfish, and *S*. *aleutianus*, the rougheye rockfish) were associated with boulders and gorgonians in Zhemchug Canyon; none were observed on Pribilof transects. *Sebastolobus alascanus*, the shortspine thornyhead, was significantly associated with gorgonian and pennatulacean corals in Zhemchug canyon, but showed no associations in Pribilof transects (Table 3). Poachers and sculpins were significantly associated with gorgonians in Pribilof Canyon. Pleuronectid flounders were not associated with corals or boulders in either canyon (Table 3).

**Table 3:** Significance values (*p* levels) for all GLM covariates.

Pribilof Canyon							
	Fish taxa						
covariate	*Sebastes alutus*	*Sebastes* spp.	*Sebastolobus alascanus*	Total rockfish	Agonidae	Cottidae	Pleuronectidae
depth	−0.01 (0.003)**<0.0001**	-	0.02 (0.006)**<0.0001**	−0.004 (0.002)**<0.0001**	0.0007 (0.004) **0.001**	0.18	0.01 (0.003)**<0.0001**
boulders	2.96 (0.9) **0.0007**	-	1.00	2.72 (0.9) **0.001**	1.00	0.67	1.00
gorgonian corals	1.12 (0.3) **0.0003**	-	1.00	0.88 (0.3) **0.001**	−3.00 (1.5) **0.04**	1.63 (0.8) **0.04**	0.42
pennatulacean corals	0.65	-	0.67	0.14	0.75	0.17	0.16
sponges	0.74 (0.3) **0.01**	-	1.00	0.45 (0.3) **0.04**	0.27	0.71	0.48

Zhemchug Canyon							
	Fish taxa						
covariate	*Sebastes alutus*	*Sebastes* spp.	*Sebastolobus alascanus*	Total rockfish	Agonidae	Cottidae	Pleuronectidae
depth	0.01 (0.01)**<0.002**	−0.001 (0.01) **0.001**	0.01 (0.006) **0.0002**	0.007 (0.004) **0.0002**	0.003 (0.003) **0.001**	0.33	0.001 (0.003) **0.001**
boulders	1.00	7.71 (1.6)**<0.0001**	1.00	6.54 (1.6)**<0.0001**	1.00	0.85	1.00
gorgonian corals	1.00	2.27 (0.8) **0.03**	2.48 (0.5)**<0.0001**	2.26 (0.5)**<0.0001**	0.11	0.22	0.20
pennatulacean corals	3.61 (0.9) **0.003**	0.32	2.00 (0.7) **0.04**	2.07 (0.5) **0.0007**	0.37	0.11	0.37
sponges	1.00	1.00	0.44	0.22	0.91	0.53	1.00

Parameter values (standard error) are given above bolded *p* values for significant covariates. *Sebastes* spp. includes *S. borealis*, the shortraker rockfish, and *S. aleutianus*, the rougheye rockfish.

Fish, coral, sponge and boulder data were analyzed as presence-absence. Transect data were pooled. Each fish taxa or category was analyzed separately. Significance was evaluated at α = 0.05. Fish taxa from Table 2 not presented here exhibited no significant relationships with any covariate.

**Figure 4:**
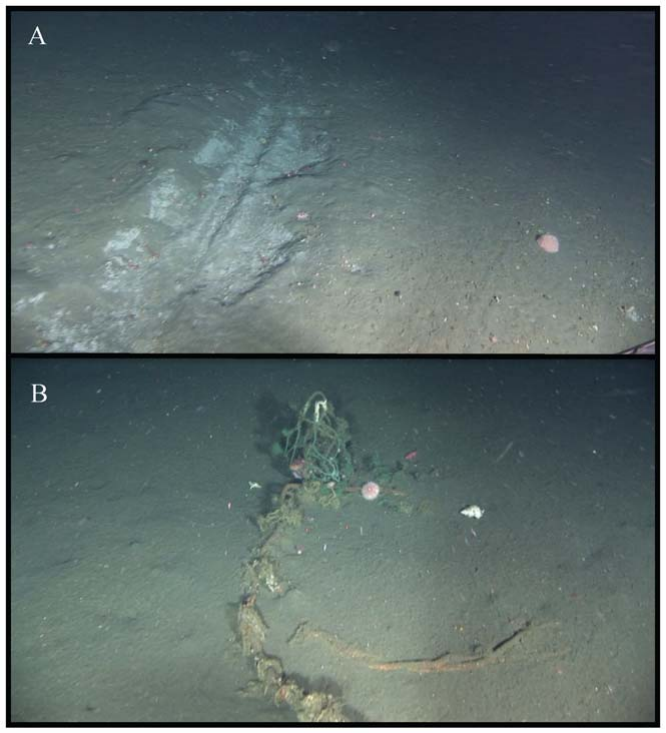
Examples of fishing damage to benthos in Pribilof Canyon, Bering Sea. A) trawl scar, 264 m depth, B) derelict fishing gear entangled on corals, 405 m depth.

Evidence of fishing disturbance was observed on a total of 13 occasions, (9 in Pribilof Canyon, 4 in Zhemchug Canyon) at depths of 154–966 meters (Table 4). Three of the 16 Deep-Worker transects had obvious evidence of fishing damage, which was recorded present in 0.26% of frames, covering 28.8 m^2^. Additional observations of damage were made on collecting dives and ROV transects (Table 4). Most observations were trawl scars evident due to gouging of soft sediments (Figure 4A). In some cases, damage to corals was evident, e.g. in Pribilof Canyon, at 280 m depth, in the form of trawl scars on the seafloor and numerous gorgonians and the pennatulacean *Halipteris willemoesi* toppled and all lying in the same direction on the seafloor. Other evidence of fishing damage included debris such as large tangles of line and netting (e.g. Figure 4B).

**Table 4:** Locations and depths of benthic fishing damage observed at Pribilof and Zhemchug Canyons.

Date	Damage type	Canyon	Depth (m)	vehicle	position
7/29/2007	debris (line)	Pribilof	410	DW	N 55°52.5718 W 168°56.4128
7/30/2007	trawl scar	Pribilof	275	DW	N 56°09.5988 W 168°48.6511
8/2/2007	trawl scar	Pribilof	336	DW	N 55°59.9834 W 169°40.6453
8/2/2007	trawl scar	Pribilof	264	DW	N 55°59.9834 W 169°40.6453
8/2/2007	trawl scar	Pribilof	284	DW	N 55°59.9834 W 169°40.6453
8/2/2007	trawl scar	Pribilof	860	ROV	N 55°57.716 W 169°39.965
8/2/2007	trawl scar	Pribilof	852	ROV	N 55°57.716 W 169°39.965
8/2/2007	debris (chain, line)	Pribilof	821	ROV	N 55°57.716 W 169°39.965
8/4/2007	debris (net)	Zhemchug	966	ROV	N 57°50.47 W 174°17.64
8/6/2007	trawl scar	Zhemchug	154	ROV	N 57°50.6 W 174°17.7
8/8/2007	debris (cable)	Zhemchug	161	ROV	N 57°51.67 W 173°50.14
8/8/2007	debris (line)	Zhemchug	317	ROV	N 58°10.08 W 174°10.51

Coordinates represent the initial starting position of each dive. DW = Deep Worker.

## Discussion

Submarine canyons have been identified as potential hotspots of deep-water biomass and productivity due to enhanced substrate and topographic complexity compared to slope habitats, as well as topographic enhancement of flux and deposition of organic matter [40]. In one of the few biological studies comparing shelf areas to a deep-sea canyon, De Leo et al [40] measured infaunal biomass in Kaikoura Canyon off New Zealand that was 100-fold higher than ever measured for a non-chemosynthetic deep-sea benthic habitat, and 10 times higher than nearby shelf benthos. Zhemchug and Pribilof Canyons lie in the highly productive “green belt” along the Bering Sea shelf edge [41], and primary production over the canyons is stimulated by stationary mesoscale eddies that enhance upwelling and can temporally extend spring phytoplankton blooms [42]. Surface-derived particulate organic carbon (POC) comprised the main source of carbon for deep-water corals, as revealed by stable isotope analyses [43], [44]. Thus, upwelling along the shelf edge and resultant high flux of phytodetritus to the seafloor, combined with the availability of hard substrate (albeit limited) on canyon slopes, likely sustains the high densities of corals and sponges measured in this study.

Abundance data for deep-water corals are scarce and are most often only reported as present or absent. Highest densities of corals within the depth range investigated were found at depths between 200–400 m, similar with other studies of corals in that depth range [11], [26], [45]. Overall mean coral density estimated in both canyons was 0.43 colonies m^−2^, approximately one third the density measured in the central Aleutian Islands via a census [11]. Density in the two canyons, however, differed considerably; Pribilof Canyon had coral densities estimated at 0.97 colonies m^−2^ only somewhat lower than that measured in the Aleutian Islands (1.23 colonies m^−2^). Coral densities estimated at Zhemchug Canyon were much lower (0.18 colonies m^−2^), but similar or higher than those observed in other coral-rich areas of the world (e.g. the Weddell Sea, Antarctica (0.12 colonies m^−2^ [46],), Atlantic Canada (0.005–0.048 colonies m^−2^ [45]), and Norway (0.043–.069 colonies m^−2^ [25]), indicating that both canyons support significant deep-water coral habitats. The somewhat deeper average depth of the Zhemchug Canyon transects may have influenced the lower coral density found there.

The overall density of corals estimated for the Bering Sea canyons are low compared to those measured via a census in the central Aleutian Islands [11] and the habitats formed by the corals are very different in the two regions (i.e. dense coral and sponge gardens were not observed in the canyons). The fields of gorgonians and pennatulaceans observed in the canyons are quite patchy in distribution and separated by large areas of open silt/sand habitat (85% of the total frames contained no corals). Nonetheless, however, the habitats formed by the corals and sponges appear to be utilized by many of the fish species present in the Canyons. Aleutian Island coral gardens (sensu [11]) have extremely high local densities of corals [11], more than three times the overall density of Pribilof Canyon, and are also particularly species rich (more than 40 species total). By contrast, we observed 15 total coral species in the Bering Sea Canyons. However, our collections (and photo identifications) included many northern range extensions and new records for the region, indicating that the canyons are more species rich than previously known [47]. Our collections included one scleractinian (*Caryophyllia alaskensis* – a northern range extension), one antipatharian (*Bathypathes* sp. – northernmost record in the Pacific Ocean), one alcyonacean (*Anthomastus* sp. – likely representing a northern range extension), one stoloniferan (*Clavularia* sp. – a new record for the region), seven gorgonians (*Plumarella superba*, *P*. *echinata*, *Primnoa pacifica*, *P*. *wingi*., *Keratoisis* sp., *Swiftia pacifica*, and *Paragorgia arborea* – several of which represent new records for the region and northern range extensions), and three pennatulaceans (*Halipteris willemoesi*, *Protoptilum* sp., and cf. *Pennatula* sp. – the latter two taxa representing probable range extensions).

Abundance of hexactinellid sponges in Pribilof Canyon was similar to that of the corals at 0.40 m^−2^; unfortunately few sponge abundance data from other areas are available for comparison. In the Eastern Gulf of Alaska, large sponges were found at mean densities of 0–0.12 m^−2^ in untrawled areas; experimental trawling significantly reduced sponge densities [48]. Deep-water bioherms off the Pacific Coast of Canada harbored abundant (11.6–26%) cover of hexactinellid sponges [49]. Density of the hexactinellid sponge *Pheronema carpenteri* in the Porcupine Seabight (Northeast Atlantic), averaged 0.34 m^−2^ at 1000–1300 m depth, with local aggregations attaining higher densities [50]. Density of *Hyalonema* sp. averaged 0.01 m^−2^ at 4100 m depth off California, but densities of dead hexactinellid stalks were much higher and harbored a high-diversity epifaunal community [12]. Overall, the hexactinellid sponge densities observed in this study were comparable to those reported as high density in areas elsewhere.

Fishes, particularly rockfishes, were associated with corals significantly more often than would be expected given the abundance of corals, a result consistent with other studies of Alaskan corals [11], [27], [51]. Rockfishes were also, however, significantly associated with boulders, and it is unclear at present whether corals generally serve as hard structure interchangeably with rocks and other forms of structure, or provide added fitness benefits to fishes such as food sources [20]. Both corals and sponges harbor diverse and abundant assemblages of macroinvertebrates that may be prey for fishes [8], [12], [52]. Pacific ocean perch was found preferentially inhabiting flat pebble bottoms in the eastern Gulf of Alaska [53]. In that study, presence of corals was not evaluated as a factor influencing fish distribution, although we note that the photo of *S. alutus* in the paper shows the fish among a dense grove of the pennatulacean *Halipteris willemoesi* [53]. Later, Brodeur [54] observed aggregations of Pacific ocean perch associated with *H. willemoesi*, and suggested that the sea pens filled the need for vertical structure for this fish species. Our results support this observation, but do not provide evidence for the relative importance of corals, sponges, and other biogenic structure versus nonbiogenic structure, e.g. boulders, to these fishes. Nevertheless, boulders, seamounts, and other sources of high-profile vertical relief are relatively rare in the deep sea [55] and abundant corals likely provide important fish habitat in low-relief areas even if their habitat quality does not differ from that of abiotic structure.

Pacific ocean perch is a late-maturing, slow-growing viviparous species and were historically the target of an important trawl fishery in the eastern Bering Sea until stocks were severely depleted about a decade ago [56]. Stocks have since increased and recently a small directed fishery has been reopened, and Pacific ocean perch continue to be taken as bycatch by other trawl fisheries. Dispersal of Pacific ocean perch is apparently quite limited, with genetic structure evident at geographic scales of 70–400 km, indicating that management of this species should be on a finer geographic scale than the current assessment areas [57]. Pacific ocean perch populations in areas such as the Canyons studied here, therefore, may be rendered less resilient if local habitats are degraded by benthic fishing impacts.

An ecosystem approach to management [58], [59], which recognizes that the value of intact ecosystems is greater than the sum of their parts [60], is now the general, though sometimes necessarily imprecise, goal of fisheries management [61]. Witherell et al [62] summarized progress towards, and future implementation of, an ecosystem approach to management of Alaskan groundfish fisheries by the North Pacific Fishery Management Council. This included, as required under the 1996 Magnuson–Stevens Act amendments, identification and conservation of essential fish habitat (EFH), as well as habitat areas of particular concern (HAPC), which are specific EFH's distinguished by their particularly important ecological function and vulnerability to anthropogenic impacts [63]. Corals were used as the *prima facie* example of a HAPC in Alaskan waters [28], [62]. Our results support this notion, indicating that commercially important fishes preferentially utilize corals for habitat. Although some of these fishes also use boulders, boulders and corals are typically associated [64], and together they comprise inseparable elements of the habitat. Although it is of ecological and evolutionary interest to separate the relative fitness values of these different forms of habitat, from a resource management standpoint, a conservative approach suggests that they be considered at least equivalent. Like most deep ocean habitats, these canyons are dominated by low-relief soft substrate, making corals important habitat elements providing vertical relief. Thus, based on the survey data reported here, Pribilof and Zhemchug Canyons can be regarded as harboring areas of high densities of slow-growing corals that form the foundation of complex communities as well as habitat areas of particular concern due to their vulnerability and use by commercially important fishes.

Deep-water gorgonian corals grow slowly and can live for hundreds of years [65], [66], [67], making likely recovery times after disturbance very long [68]. Anthropogenic disturbance by bottom trawling has been shown to devastate corals, not surprisingly, since bottom trawls typically incorporate heavy chains and doors that are dragged along the seafloor [69]. Heavily trawled areas in the Aleutian Islands were devoid of hydrocorals, and levels of damage to corals were positively correlated with trawling effort [70]. Bottom longline and pot gear can also damage corals, although this damage is likely less intense than that from trawling [11]. In this study, fishing damage was observed on several dives (Figure 4, Table 4); suggesting that bottom-contact fishing is clearly a source of disturbance to corals in Pribilof and Zhemchug Canyons. The high densities of corals in these shelf-edge canyons, and their use as fish habitat, suggests that conservation of these unique areas be given priority status in fisheries management decisions. Canyons and other topographic features on the edges of continental shelves likely represent high productivity areas throughout the deep sea [40], and the ecology and conservation of these habitats and the ecosystem services they provide deserve more attention.

## Supporting Information

Figure S1 **Screenshot of Digital notebook while in graphical annotation mode.**
(TIF)

Text S1 **Supplementary material describing access to annotated data, extraction and annotation process.**
(DOCX)
