# Supplementary figures and images for: Structure-Forming Corals and Sponges and Their Use as Fish Habitat in Bering Sea Submarine Canyons
Source: PLoS One. 2012 Mar 21;7(3):e33885. doi: 10.1371/journal.pone.0033885 (PMC3309998; doi:10.1371/journal.pone.0033885)

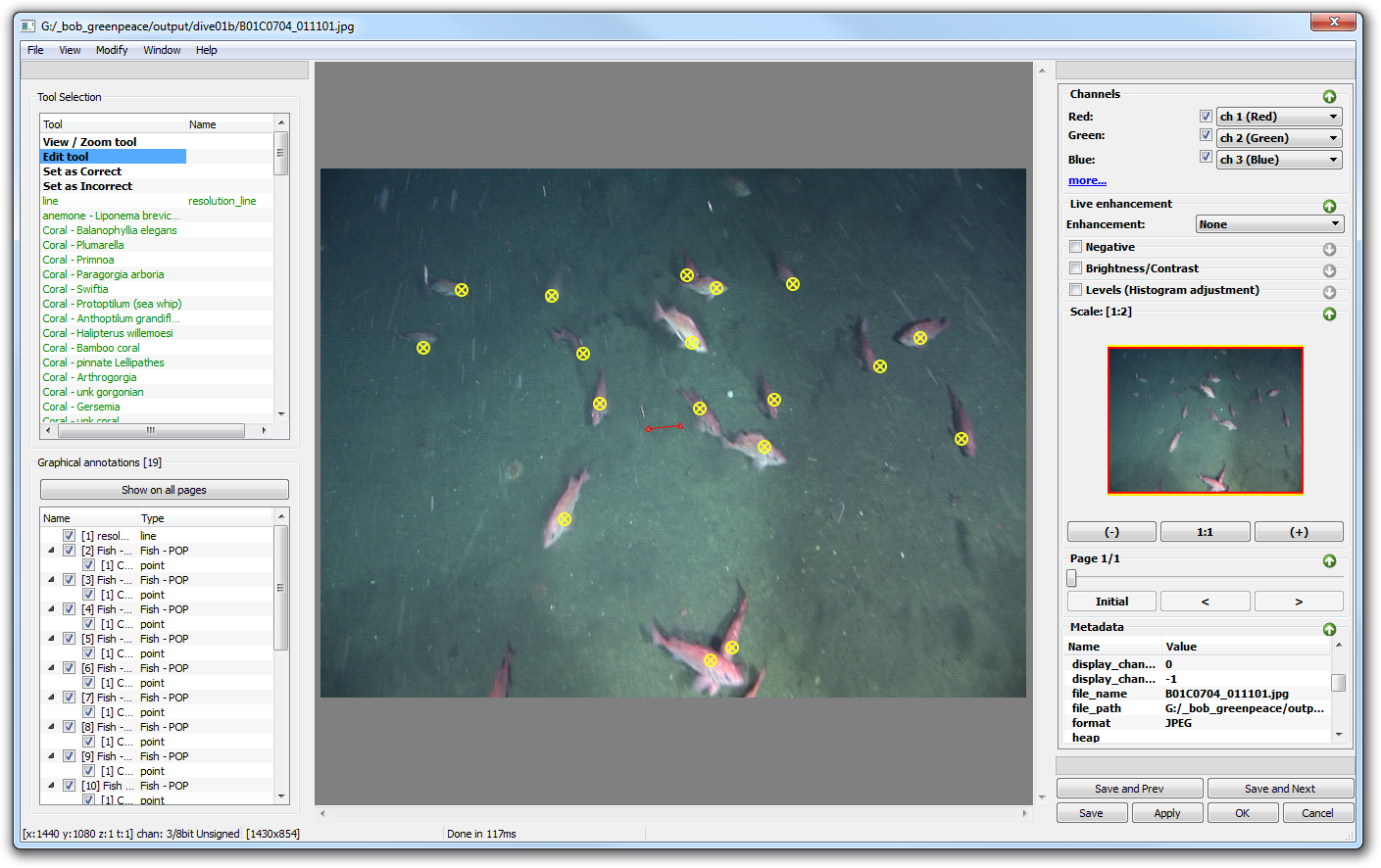

Supplement: Figure S1 — Screenshot of Digital notebook while in graphical annotation mode. (TIF) [file pone.0033885.s001.tif]
