# Supplementary material for: Structure-Forming Corals and Sponges and Their Use as Fish Habitat in Bering Sea Submarine Canyons
Source: PLoS One. 2012 Mar 21;7(3):e33885. doi: 10.1371/journal.pone.0033885 (PMC3309998; doi:10.1371/journal.pone.0033885)
Supplement: Text S1 — Supplementary material describing access to annotated data, extraction and annotation process. (DOCX) [file pone.0033885.s002.docx]

# Structure-forming corals and sponges and their use as fish habitat in Bering Sea submarine canyons

# Supplementary material

# 1 Access to original results and data

All of the original images along with all the data necessary for this analysis, automatic laser dot detection and manual annotations are available from our database:

<http://bisque.ece.ucsb.edu/client_service/view?resource=http://bisque.ece.ucsb.edu/data_service/dataset/1580770>

In addition to the reach web interface where one can survey the annotations and search for images, all data can be accessed via the XML format. You can access the references to images via a dataset:

<http://bisque.ece.ucsb.edu/data_service/datasets/1580770?view=deep>

One can also make a dynamic query to obtain all the image data along with annotations in one large XML document:

[http://bisque.ece.ucsb.edu/data_service/images?tag_query=image_type:"Bering%20sea%20canyons"](http://bisque.ece.ucsb.edu/data_service/images?tag_query=image_type:%22Bering%20sea%20canyons%22)

Each image will have textual annotations like these:

<**tag** name="area" value="1.12147447759"/>
<**tag** name="depth" value="488"/>
<**tag** name="angle_pitch" value="30"/>
<**tag** name="pixel_resolution_x" value="0.00059019908412"/>
<**tag** name="video_frame" value="19201"/>
<**tag** name="video_file" value="B01C0211.M2T"/>
<**tag** name="dive_number" value="26"/>
<**tag** name="canyon_name" value="Zhemchug"/>
<**tag** name="image_type" value="Bering sea canyons"/>

One can notice that the annotation "image_type" was used to make the query. It is also possible to compose a query finding all images of the dive #26 and frame number 19201 and additionally get all the annotation data associated with these images:

<http://bisque.ece.ucsb.edu/data_service/images?tag_query=image_type:%22Bering%20sea%20canyons%22%20AND%20dive_number:26%20AND%20video_frame:19201&view=deep>

The data also contains graphical annotations in the form:

<**gobject** name="Fish - poacher" type="Fish - poacher">

<**point** name="Centroid" type="point">

<**vertex** y="1042.0" index="0" x="706.0"/>
 </**point**>
</**gobject**>

<**polyline** name="resolution_line" type="polyline">
 <**vertex** y="348.0" index="0" x="702.0"/>
 <**vertex** y="370.0" index="1" x="828.0"/>
</**polyline**>

Here the former polyline describes detected laser dots and is used to estimate the pixel resolution at the center of the image.

All other objects are described by their centroid position as is in the first object. We have defined 54 different types of annotations that can be found in this data.

# 2 Frame extraction from HD video

For the task of frame extraction we have used the Bio-Image Convert command line software:

<http://bioimage.ucsb.edu/downloads/BioImage%20Convert>

It can decode most HD video formats and allows sampling of frames. Another useful feature is the ability to detect overlap between the frames and skip the frames with large overlap. This feature uses advanced image registration and thus slows the overall processing time. An example command line call to extract frames from the B01C0803.M2T file with minimum allow overlap of 5%:

imgcnv -i B01C0803.M2T -o B01C0803.jpg -t jpeg -sampleframes 300 -no-overlap 5

# 3 Annotation with the Digital Notebook

Manual annotations for this study were done using the Digital Notebook software:

<http://bioimage.ucsb.edu/downloads/Digital%20Notebook>

This software allows a rapid and configurable textual and graphical annotation of many images. It's possible to create templates to add types of species of interest and also define what graphical primitive will be used. We have created specific templates for the purposes of our study:

<http://bioimage.ucsb.edu/beringcanyons/>[digital_notebook_templates.zip](http://bioimage.ucsb.edu/beringcanyons/digital_notebook_templates.zip)

An example screenshot of an image annotation process is shown on Figure 1.

| 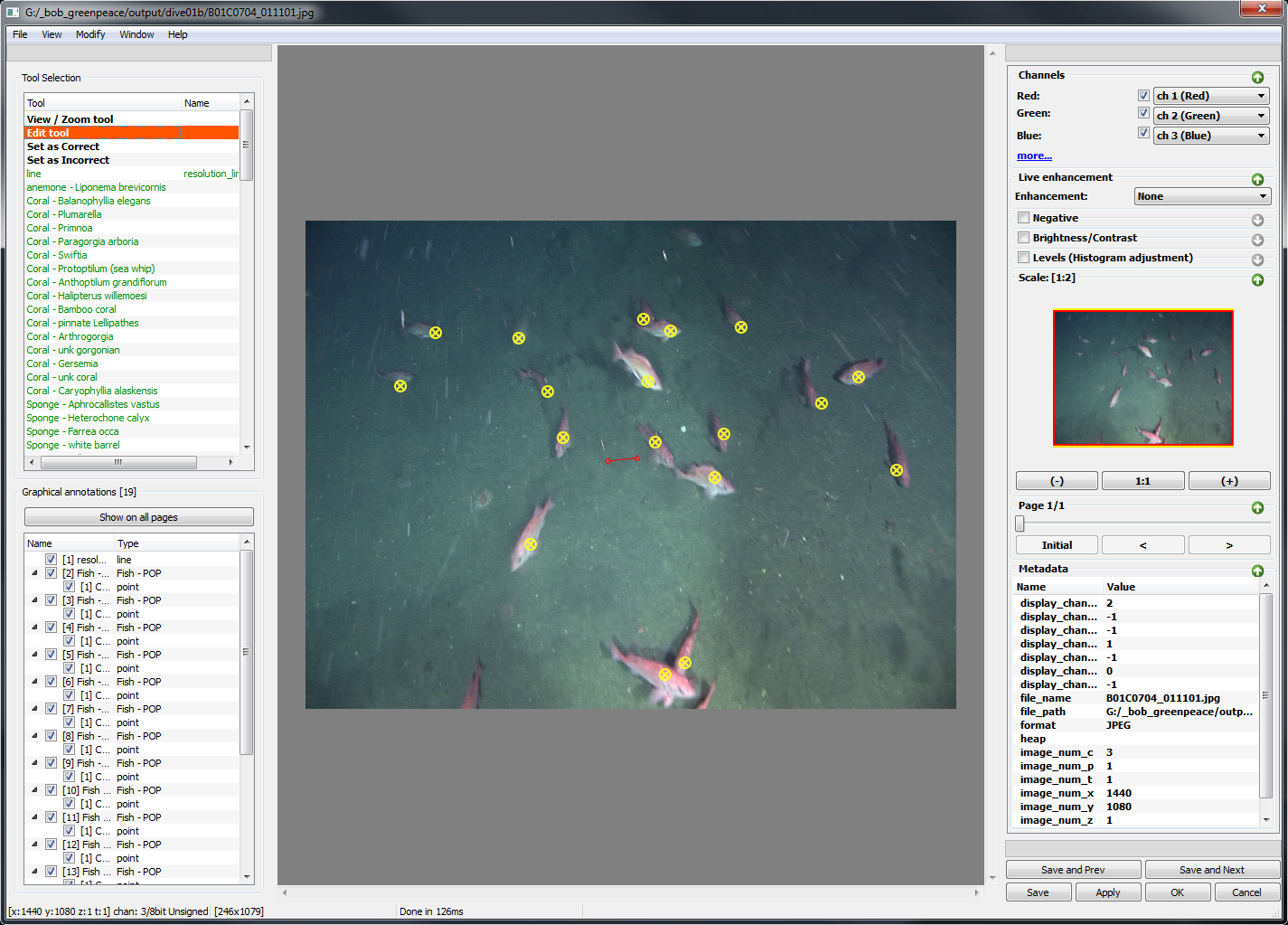 |
| --- |
| Figure 1. Screenshot of Digital notebook while in graphical annotation mode |
